# Computational design of single-stranded DNA hairpin aptamers immobilized on a biosensor substrate

**DOI:** 10.1038/s41598-021-88796-2

**Published:** 2021-05-26

**Authors:** Iman Jeddi, Leonor Saiz

**Affiliations:** Modeling of Biological Networks and Systems Therapeutics Laboratory, Department of Biomedical Engineering, University of California, 451 East Health Sciences Drive, Davis, CA 95616 USA

**Keywords:** Computational biophysics, DNA nanostructures

## Abstract

Aptamer interactions with a surface of attachment are central to the design and performance of aptamer-based biosensors. We have developed a computational modeling approach to study different system designs—including different aptamer-attachment ends, aptamer surface densities, aptamer orientations, and solvent solutions—and applied it to an anti MUC1 aptamer tethered to a silica biosensor substrate. Amongst all the system designs explored, we found that attaching the anti MUC1 aptamer through the 5′ terminal end, in a high surface density configuration, and solvated in a 0.8 M NaCl solution provided the best exposure of the aptamer MUC1 binding regions and resulted in the least amount of aptamer backbone fluctuations. Many of the other designs led to non-functional systems, with the aptamer collapsing onto the surface. The computational approach we have developed and the resulting analysis techniques can be employed for the rational design of aptamer-based biosensors and provide a valuable tool for improving biosensor performance and repeatability**.**

## Introduction

Aptamers are short oligonucleotides with high affinity binding to specific molecular targets. The interest of aptamers as key elements in biosensors results from the conformational changes during the process of aptamer and target interaction that can lead to detectable signals^1^. They have found multiple uses in biosensing because of their robustness and simplicity, and they are involved in multiple direct detection strategies. Yet, aptamer-based biosensors need to be optimized for consistency and reproducibility to become commercially feasible. Success in this direction would greatly benefit from a detailed molecular-level understanding of the processes involved^2^. In particular, computational approaches provide an avenue to complement and guide experimental studies for advancing our understanding of these biomolecular complexes at multiple levels, including their structure, dynamics, molecular interactions, and solvent effects^3–7^.

Because of its high relevance, we focus on the Mucin 1 (MUC1) binding aptamer tethered to a silica biosensor substrate. Mucin 1 protein is a well-studied biomarker^8^ for the early detection of epithelial cancer^9^. In breast and ovarian carcinomas, the MUC1 protein is overexpressed and free floating in the bloodstream^9^ and is a good target for the development of early stage cancer diagnostic tests. Using the SELEX process^10^, identification of an aptamer that binds with high affinity and selectivity to the MUC1 protein has been reported^11^. In addition, the three dimensional structure for the anti MUC1 aptamer has been resolved experimentally and is available through the Protein Data Bank (PDBID 2L5K)^12^. Specifically, the identified anti MUC1 aptamer is a 23-nucleotide DNA hairpin structure with a three-nucleotide thymine loop (CAGTTGATCC**TTT**GGATACCCTG).

Experimental studies of the anti MUC1 aptamer have indicated strong binding of the aptamer to certain exposed peptides (APDTRPAPG) within the mucin 1 protein^11^. While these experimental studies have shown high binding affinity of the identified anti MUC1 aptamer to the MUC1 peptide, details of the binding process including the aptamer orientation and location of the binding sites between the MUC1 peptides and the aptamer were not resolved in detail.

In order to gain further insights into the peptide-aptamer binding characteristics of the anti MUC1 aptamer and the MUC1 protein, Rhinehardt et al.^13^ conducted multiple all atom molecular dynamics simulation studies using the anti MUC1 aptamer and the MUC1 peptide in solution. These studies indicated consistent binding of the MUC1 peptide with the thymine loop of the aptamer (TTT) initiated by the arginine residue of the peptide^13^. In similar studies, atomistic molecular dynamic simulations on single and double mutants of the anti MUC1 aptamer showed increased affinity to the MUC1 ligand^14^.

The molecular dynamics studies conducted by Rhinehardt et al. have provided important information regarding the binding site location and orientation of the anti MUC1 aptamer and the MUC1 protein when solvated in an aqueous environment. While the results of Santini et al. provided further details on mutants of the anti MUC1 aptamers and its relationship with binding free energies^14^. In particular, they showed that the double mutant aptamer exhibits a tight interaction with the MUC1 peptide and adopts a groove conformation that structurally favors the intermolecular contact with the peptide leaving the 3′ and 5′ ends free for further chemical conjugation^14^.

Nevertheless, experimental and computational studies have shown that the orientation^15,16^ or conformation^17^ of a tethered biorecognition element on a biosensor surface can change upon immobilization and can directly impact the performance of the biosensor. For example, typically aptamers are attached to the biosensor substrate through the 5′ end^18^. These examples encompassed electrochemical sensors based on the target-induced folding or unfolding of electrode-bound oligonucleotides, such as those directed to the thrombin protein and the platelet-derived growth factor (PDGF). Yet, Revzin and coworkers demonstrated through experimental surface plasmon resonance (SPR) studies that immobilizing an interferon-gamma (IFN-gamma) binding aptamer to a biosensor substrate via the 3′ end resulted in higher binding affinities with the target when compared to attachment via the 5′ end^19^.

Herein we report the results of all atom molecular dynamics simulation studies using the anti MUC1 aptamer tethered to a biosensor substrate with the aim of understanding the aptamer-surface interactions and gaining insights into improved biosensor design. Explicitly, we studied different system designs, including different aptamer-attachment ends, aptamer surface densities, aptamer orientations, and solvent solutions of the DNA aptamer attached to a substrate. The computational approach we have developed provides a methodological framework that can generally be applied to a wide range of systems where an aptamer is attached to a biosensor surface or to a nano-delivery system, including those attached to gold surfaces as well^20^.

## Methods and computational details

### Model of the aptamer biosensor

The initial 3D model of the anti MUC1 DNA aptamer used in the computational studies was downloaded from the Protein Data Bank (PDBID 2L5K)^12^. Even though initial configurations for atomistic molecular simulations are typically based on experimentally resolved structures, in general, it would also be possible to use recently developed computational methods that allow the prediction of the 3D structure of single-stranded DNA (ssDNA) hairpins from sequence^21^. In order to investigate the orientation and conformation of the aptamer tethered on a biosensor substrate, we performed ten separate all atom MD simulation studies using ten different starting configurations. The following methods sections contain detailed descriptions of the ten different starting configurations and Table 1 contains a summary of the configurations.

**Table 1:** Different anti MUC1 DNA aptamer biosensor configurations used for the MD simulation studies.

	Aptamer Attachment End	Aptamer Starting Orientation to Biosensor Substrate	Aptamer Surface Density	Solution
Configuration 1a	5′	Parallel	Low	Neutral
Configuration 1b	5′	Parallel	Low	0.8 M NaCl
Configuration 2a	5′	Perpendicular	Low	Neutral
Configuration 2b	5′	Perpendicular	Low	0.8 M NaCl
Configuration 3a	5′	Perpendicular	High	Neutral
Configuration 3b	5′	Perpendicular	High	0.8 M NaCl
Configuration 4a	3′	Perpendicular	Low	Neutral
Configuration 4b	3′	Perpendicular	Low	0.8 M NaCl
Configuration 5a	3′	Perpendicular	High	Neutral
Configuration 5b	3′	Perpendicular	High	0.8 M NaCl

### Biosensor substrate

The biosensor substrate model consisted of a silicon dioxide (SiO_2_) crystal surface and was created using the Inorganic Builder plugin of the Visual Molecular Dynamics (VMD) program^22^. The SiO_2_ model unit size was 49.78 × 49.78 × 6.948 Å^3^ which is equivalent to a single layer of SiO_2_ arranged in a 10 × 10 crystal. The size of the substrate unit was specifically selected to enable modeling of representative experimentally derived aptamer surface densities as described below. Here we focused on a silica surface, but our approach can be extended to other type of biosensor surfaces, such as gold and glass surfaces with a different type of aptamer-surface attachments^1,2^.

### Aptamer surface density

Multiple experimental studies have reported on the effect of aptamer surface density on the sensitivity and limit of detection of biosensors^23–25^. Typically, during the biosensor manufacturing process, the aptamer surface density is controlled by employing different concentrations of the aptamer solution during the surface immobilization process. Different voltammetry methods are then used to experimentally determine the final aptamer surface density immobilized on the biosensor electrode.

Plaxco and coworkers^24^ reported the fabrication of aptamer-based cocaine and thrombin sensors using aptamer concentrations of 0.01 to 1.25 µM. Using the above noted methodology, they were able to reproducibly achieve aptamer surface densities ranging from 1.2 × 10^11^ to 4.4 × 10^12^ molecules/cm^2^ for the cocaine sensor and 5.7 × 10^11^ to 1.3 × 10^13^ molecules/cm^2^ for the thrombin sensor. For the cocaine sensor, highest sensor sensitivity was achieved at the lowest aptamer surface densities; while for the thrombin aptamer sensor, the highest sensor sensitivity was achieved at intermediate aptamer surface densities^24^.

The Revzin group^23^ reported on the fabrication of an aptamer-based sensor for the detection of IFN-gamma. Using the same methodology, aptamer solution concentrations of 0.5, 2.0, and 8.0 µM were employed to fabricate the biosensors resulting in estimated aptamer surface densities of 4.17 × 10^12^ molecules/cm^2^, 8.53 × 10^12^ molecules/cm^2^, and 6.57 × 10^13^ molecules/cm^2^, respectively. The highest sensor sensitivity was demonstrated at the lowest aptamer surface density^23^.

In order to understand the effects of immobilized anti MUC1 aptamer surface density on the conformation and orientation of the aptamer, we selected two model configurations representing "low" and "high" aptamer surface densities, that correspond to one and two molecules per simulation box, respectively. Specifically, these are characterized by:

(i) *"Low" surface density:* 1 molecule/simulation box ~ 4 × 10^–4^ molecules/Å^2^ = 4 × 10^12^ molecules/cm^2^.

(ii) *"High" surface density:* 2 molecules/simulation box ~ 8 × 10^–4^ molecules/Å^2^ = 8 × 10^12^ molecules/cm^2^.

### Aptamer attachment and surface coating

**Figure 1:**
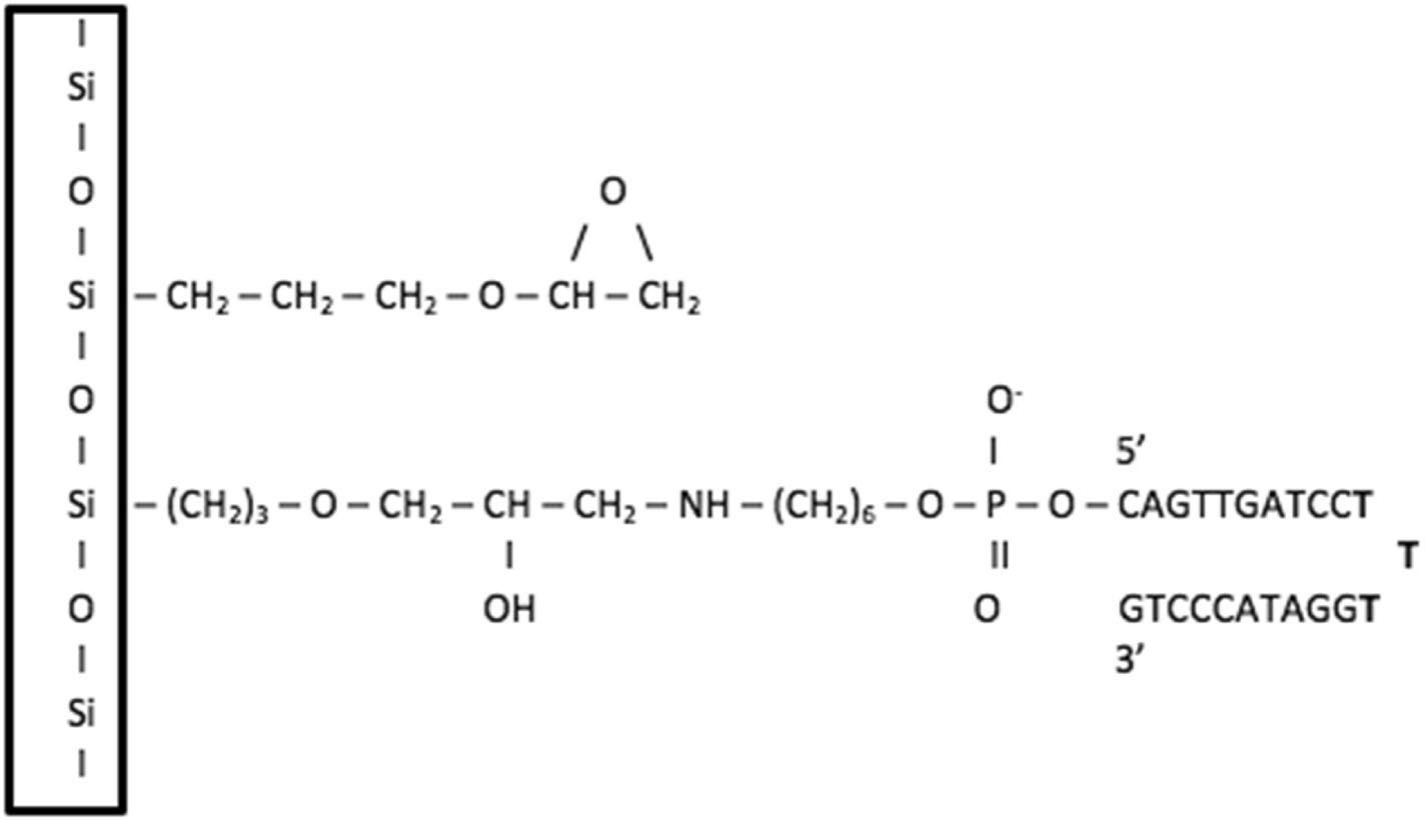
Cartoon of the chemical structure of epoxide-amine linker and epoxide monolayer attachment.(Top) Epoxide monolayer molecule attached to SiO_2_ surface (Bottom) Epoxide-amine linker molecule attached to SiO_2_ surface and 5′ terminal of the aptamer.

**Figure 2:**
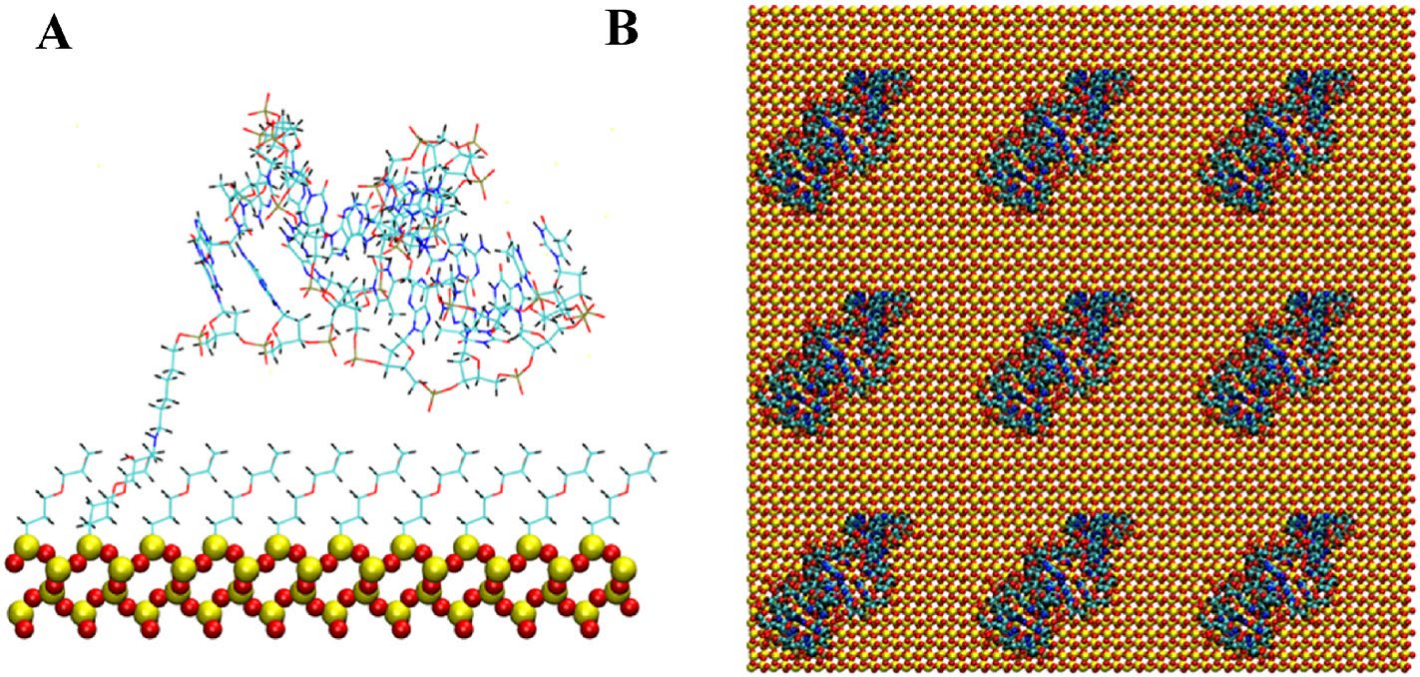
Anti MUC1 aptamer tethered to SiO_2_ biosensor substrate in configuration 1 (5′ end attachment, parallel to surface, low density). (A) Side view of simulation cell (B) Top view with periodic display. Water and ion molecules are not displayed.

**Figure 3:**
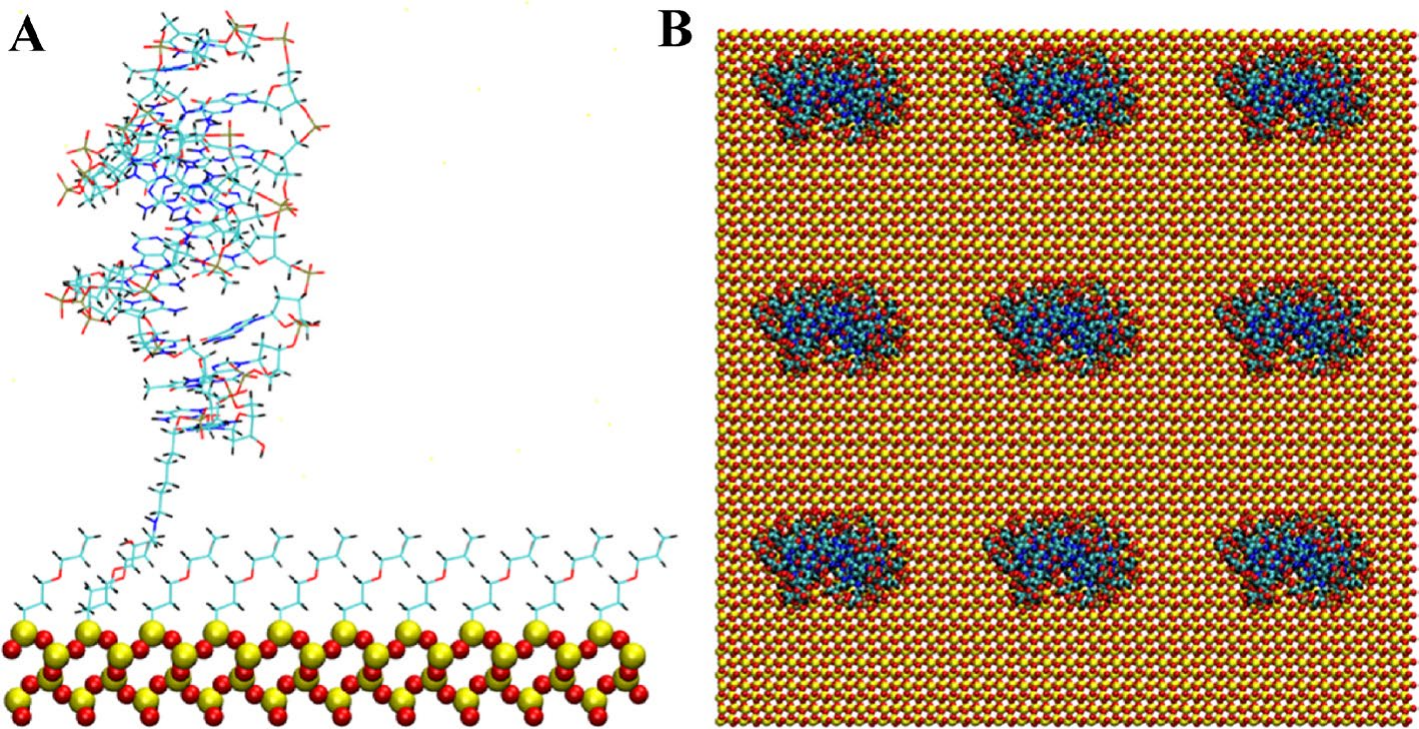
Anti MUC1 aptamer tethered to SiO_2_ biosensor substrate in configuration 2 (5′ end attachment, perpendicular to surface, low density). (**A**) Side view of simulation cell (**B**) Top view with periodic display. Water and ion molecules are not displayed.

**Figure 4:**
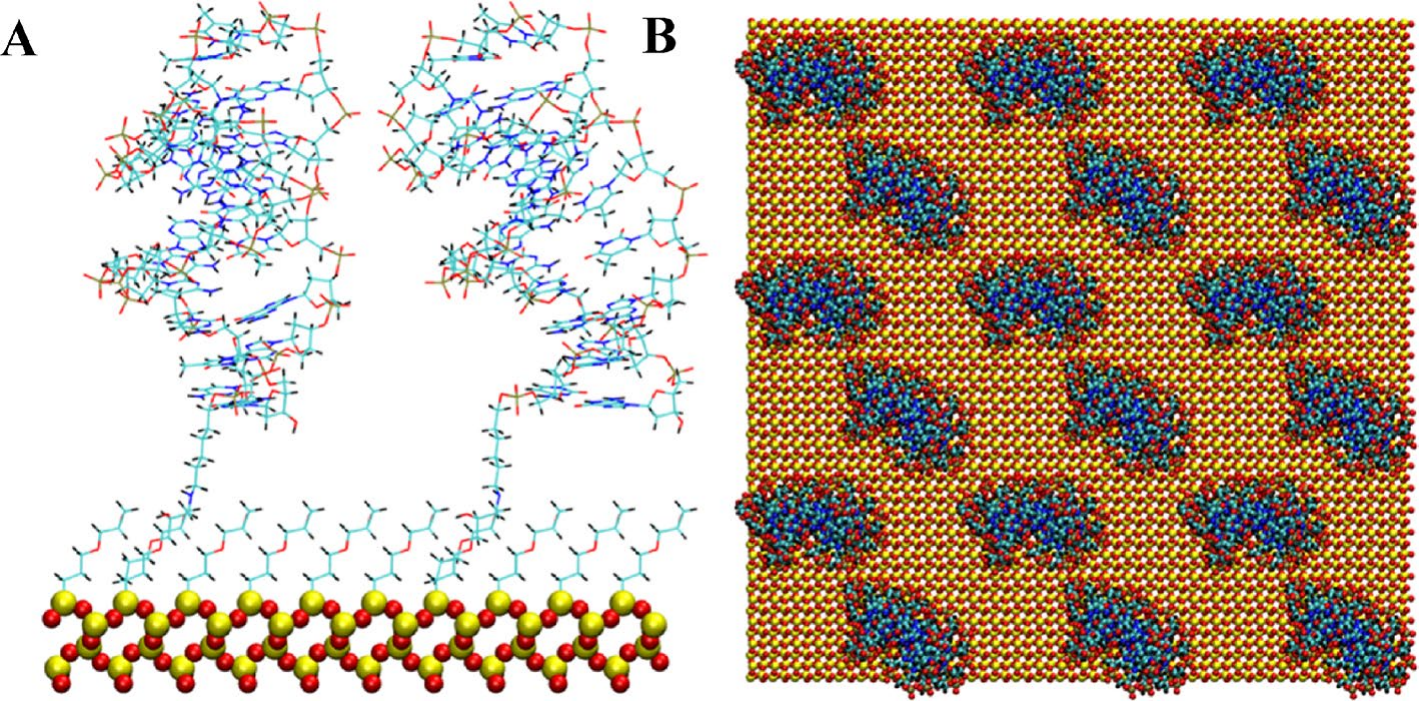
Two anti MUC1 aptamers tethered to SiO_2_ biosensor substrate in configuration 3 (5′ end attachment, perpendicular to surface, high density). (**A**) Side view of simulation cell (**B**) Top view with periodic display. Water and ion molecules are not displayed.

**Figure 5:**
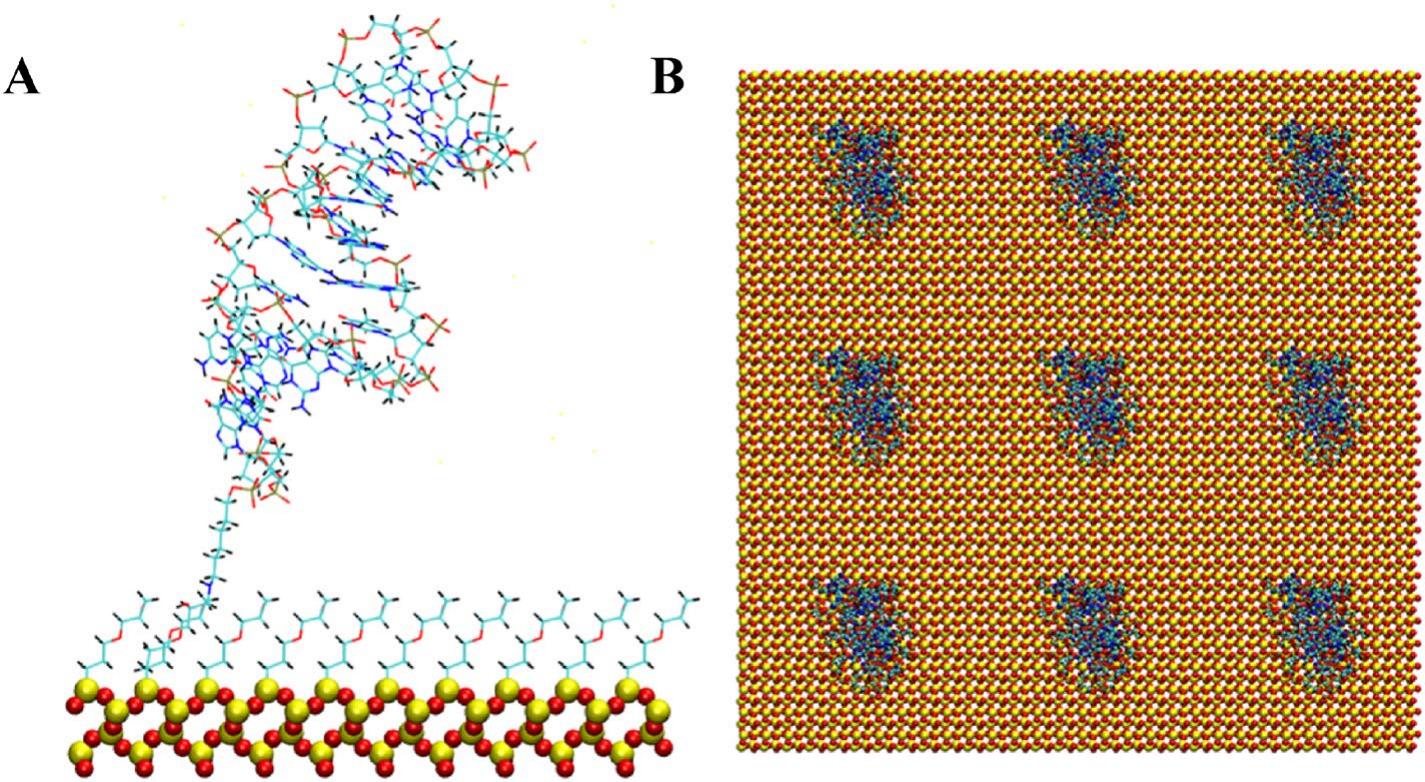
Anti MUC1 aptamer tethered to SiO_2_ biosensor substrate in configuration 4 (3′ end attachment, perpendicular to surface, low density) (**A**) Side view of simulation cell (**B**) Top view with periodic display. Water and ion molecules are not displayed.

**Figure 6:**
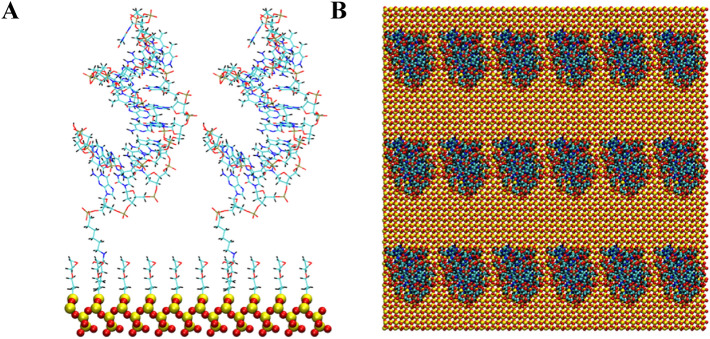
Two anti MUC1 aptamers tethered to SiO_2_ biosensor substrate in configuration 5 (3′ end attachment, perpendicular to surface, high density) (**A**) Side view of periodic cell (**B**) Top view with periodic display. Water and ion molecules are not displayed.

**Figure 7:**
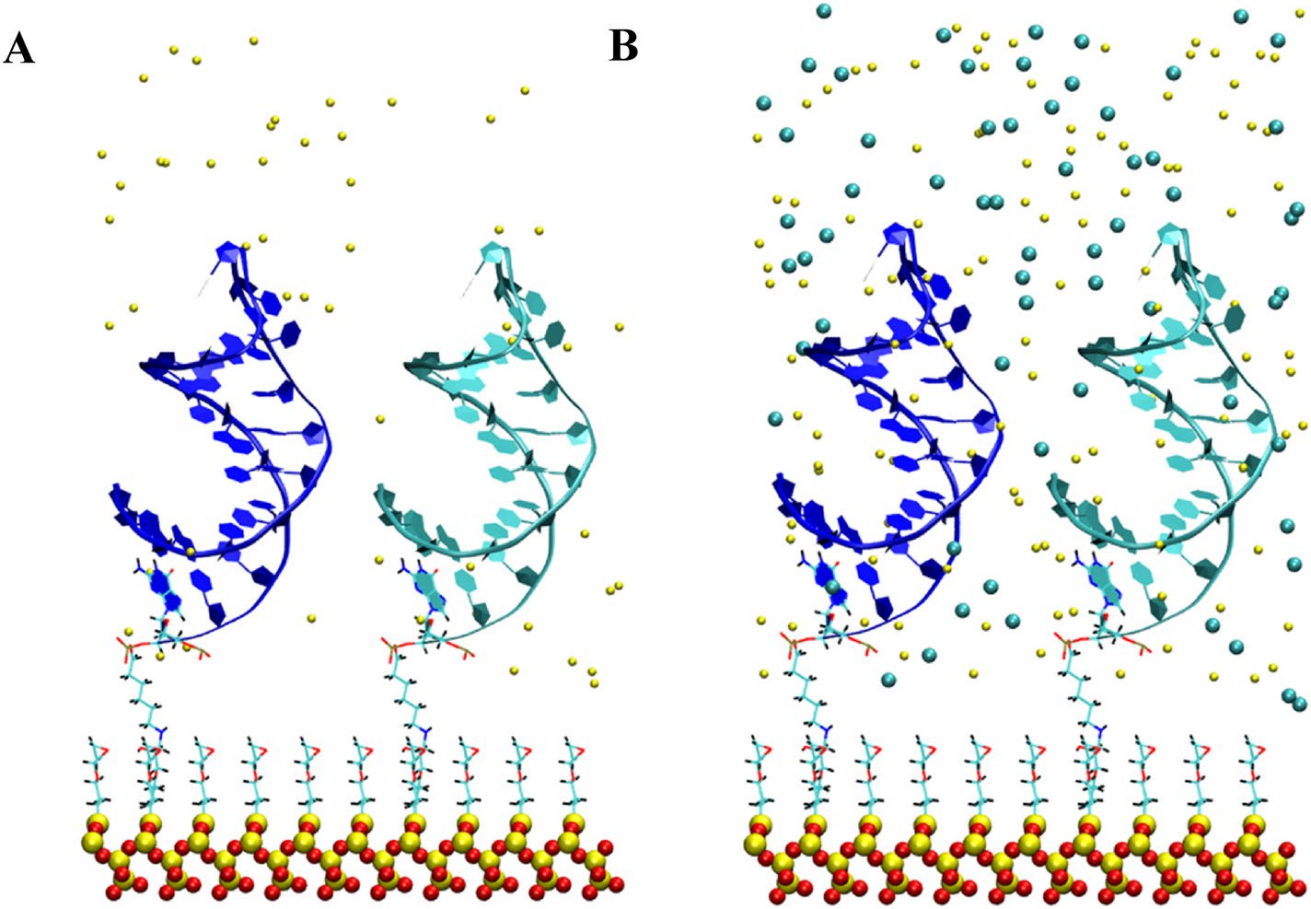
Representative starting configurations for different ion concentrations. (**A**) Configuration 5a corresponds to the neutralized system and (**B**) Configuration 5b corresponds a 0.8 M solution after neutralization. Water atoms are not displayed. Sodium ions are shown in yellow (**A**, **B**) and Chloride ions are shown in cyan (**B**).

The anti MUC1 DNA aptamer was attached from either the 5′ or 3′ terminal of the aptamer to the silica substrate using an epoxide-amine linker. The remaining surface of the silica substrate was coated with an epoxide monolayer. This aptamer attachment and surface coating is consistent with previous studies for a DNA duplex tethered to a silica surface^16^. A cartoon of the chemical structure of the epoxide-amine linker and epoxide monolayer molecules as attached to the aptamers and SiO_2_ surface is shown in Fig. 1. Figures 2, 3, 4, 5, 6 and 7 show a detailed atomistic structure of the initial configurations of the system for the different conditions.

The epoxide-amine linker and epoxide monolayer molecules were both modeled using the Molefacture plugin of the VMD program.

Both molecules were parametrized by manually creating a force field topology file containing the type, mass, charge of every atom, and bonds between atoms in the residue as detailed in the Supporting Information. The values for the topology file were derived from the CHARMM General force field (CGenFF) for Small Molecule Drug Design^26^, and the all-atom CHARMM force field for nucleic acids (CHARMM27)^27^.

Two different aptamer starting orientations where used: parallel or perpendicular to the SiO_2_ substrate.

### Solvation and solution concentration

Each model configuration was solvated in a water box using the Solvate plugin in VMD^22^. In order to understand the effects of solution concentration on the conformation and orientation of the immobilized aptamer, each system was either (a) neutralized by replacing a predetermined number of water molecules with sodium ions to achieve electroneutrality or (b) set to a concentration of 0.8 M after neutralization by replacing a predetermined number of water molecules with Na^+^ and Cl^−^ ions. Table 2 contains the dimensions of the simulation cells as well as the type and number of ions used in each system. Figures 2, 3, 4, 5, 6 and 7 contain the graphical depiction of the starting configurations. Because different viewpoints were chosen to better represent the different systems, Figs. 6 and 7 show a different orientation with respect to Figs. 2, 3, 4 and 5.

**Table 2:** Dimensions of the simulation box, number of atoms of the SiO_2_ substrate, number of aptamer molecules and atoms, number of epoxide molecules and atoms, number of epoxide-amine linkers and atoms, ion type and number of ions in the system, number of water atoms, and total number of atoms for each of the 10 different molecular dynamics simulations.

Model	*L*_X_ (Å)	*L*_Y_ (Å)	*L*_Z_ (Å)	SiO_2_ Atoms	Aptamer Molecules/Atoms	Epoxide Molecules/Atoms	Epoxide-amine Molecules/Atoms	Ion Type	No of Ions	No of Water Atoms	Total Atoms
Configuration 1a	51	52	105	1200	1/723	99/1881	1/48	Na^+^	32	19,020	22,904
Configuration 1b	51	52	105	1200	1/723	99/1881	1/48	Na^+^/Cl^−^	99/67	18,618	22,636
Configuration 2a	51	53	106	1200	1/723	99/1881	1/48	Na^+^	32	19,662	23,546
Configuration 2b	51	53	106	1200	1/723	99/1881	1/48	Na^+^/Cl^−^	102/70	19,242	23,266
Configuration 3a	53	54	106	1200	2/1446	98/1862	2/96	Na^+^	56	18,600	23,260
Configuration 3b	53	54	106	1200	2/1446	98/1862	2/96	Na^+^/Cl^−^	121/65	18,210	23,000
Configuration 4a	51	53	106	1200	1/726	99/1881	1 /45	Na^+^	22	19,704	23,578
Configuration 4b	51	53	106	1200	1/726	99/1881	1 /45	Na^+^/Cl^−^	92/70	19,284	23,298
Configuration 5a	51	53	106	1200	2/1452	98/1862	2/90	Na^+^	54	18,414	23,072
Configuration 5b	51	53	106	1200	2/1452	98/1862	2/90	Na^+^/Cl^−^	118/64	18,030	22,816

### Molecular dynamics simulation details

All the molecular dynamics simulations were carried out using the NAMD2.9 software package^28^ with the recent version of the all-atom CHARMM force field for nucleic acids (CHARMM27)^29^, the CHARMM General force field (CGenFF) for Small Molecule Drug Design^26^, and the rigid TIP3P model for water^30^. Additional force field parameters needed for interface atoms (i.e., linker atoms connecting to the DNA aptamer atoms) were derived from the above noted force fields. The Supporting Information contains a detailed list of the parameters used for the interface atoms. The SiO_2_ substrate was parameterized by creating a force field topology file containing the type, mass, charge of every atom, and bonds between atoms in the residue as detailed in the Supporting Information. The values for the topology file were derived from the Consistent Valence force field (CVFF)^31^.

Following standard procedures, the solvated systems were first minimized for 100,000 steps using the conjugate gradient energy minimization method as implemented in NAMD. The minimization step was followed by a gradual heating process using temperature increments of 10 K up to a final temperature of 300 K over 600 ps. The slow heating stage was then followed with an NPT production run of a total of 10 ns at a temperature of 300 K and a pressure of 1 atm. To maintain these conditions, the Langevin dynamics method was used with a friction constant of 1* ps*^-1^ and the Nose–Hoover Langevin piston method^32^. The simulations were carried out using a time step of 2 fs. In all the simulations, the SiO_2_ atoms were constrained and three-dimensional periodic boundary conditions with the minimum image convention^33^ were used to calculate the short-range Lennard–Jones interactions using a spherical cutoff distance of 12 Å with a switch distance of 10 Å. The long-range electrostatic interactions were calculated by using the particle-mesh Ewald (PME) method^34^. Details of the simulations are shown in Table 2.

## Results and discussion

### Aptamer orientation during the MD simulations

**Figure 8:**
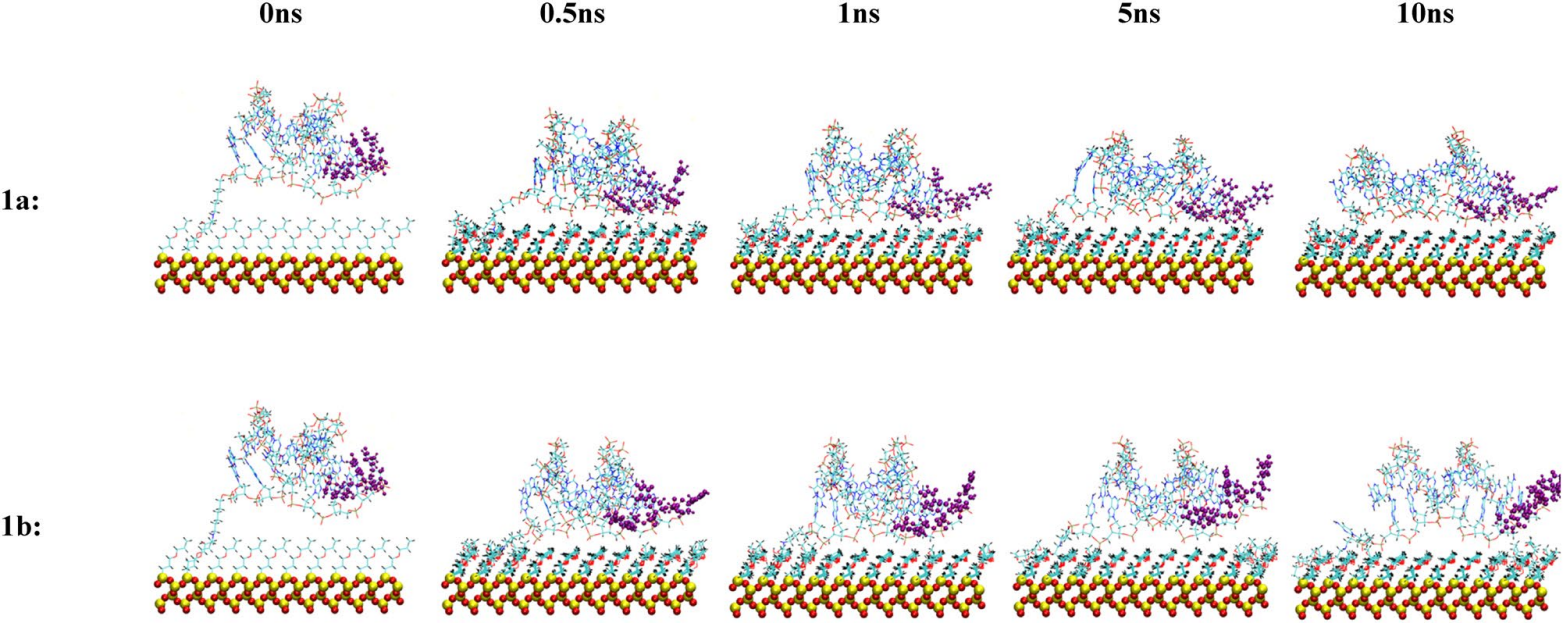
Snapshots of the MD simulation of the anti MUC1 aptamer tethered to the SiO_2_ biosensor substrate in configuration 1 (5′ end attachment, parallel to surface, low density) neutralized (top) and in 0.8 M (bottom) solution concentrations at 0 ns, 0.5 ns, 1 ns, 5 ns, and 10 ns. The MUC1 binding residues (thymine residues 11 and 13) are displayed in purple.

**Figure 9:**
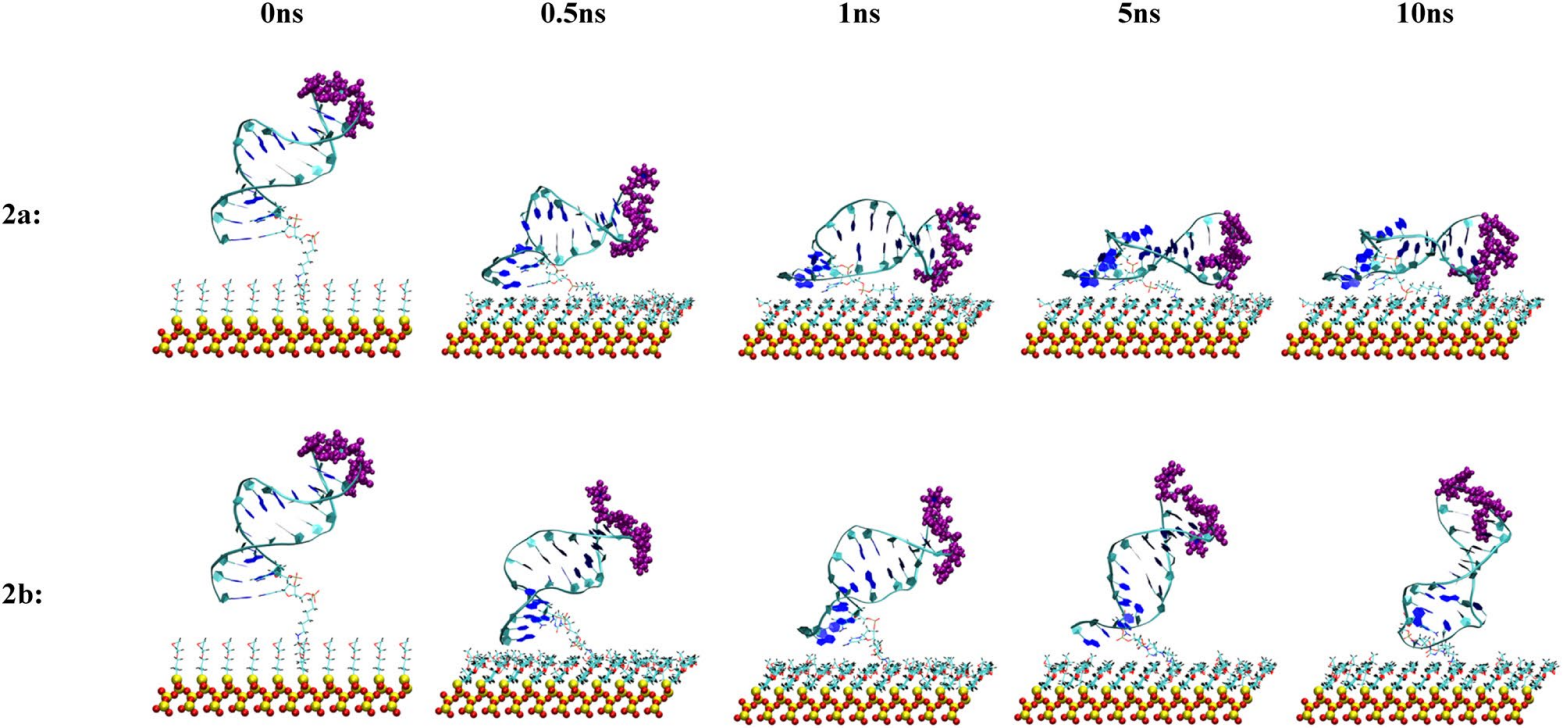
Snapshots of the MD simulation of the anti MUC1 aptamer tethered to the SiO_2_ biosensor substrate in configuration 2 (5′ end attachment, perpendicular to surface, low density) neutralized (top) and in 0.8 M (bottom) solution concentrations at 0 ns, 0.5 ns, 1 ns, 5 ns, and 10 ns. The MUC1 binding residues (thymine residues 11 and 13) are displayed in purple.

**Figure 10:**
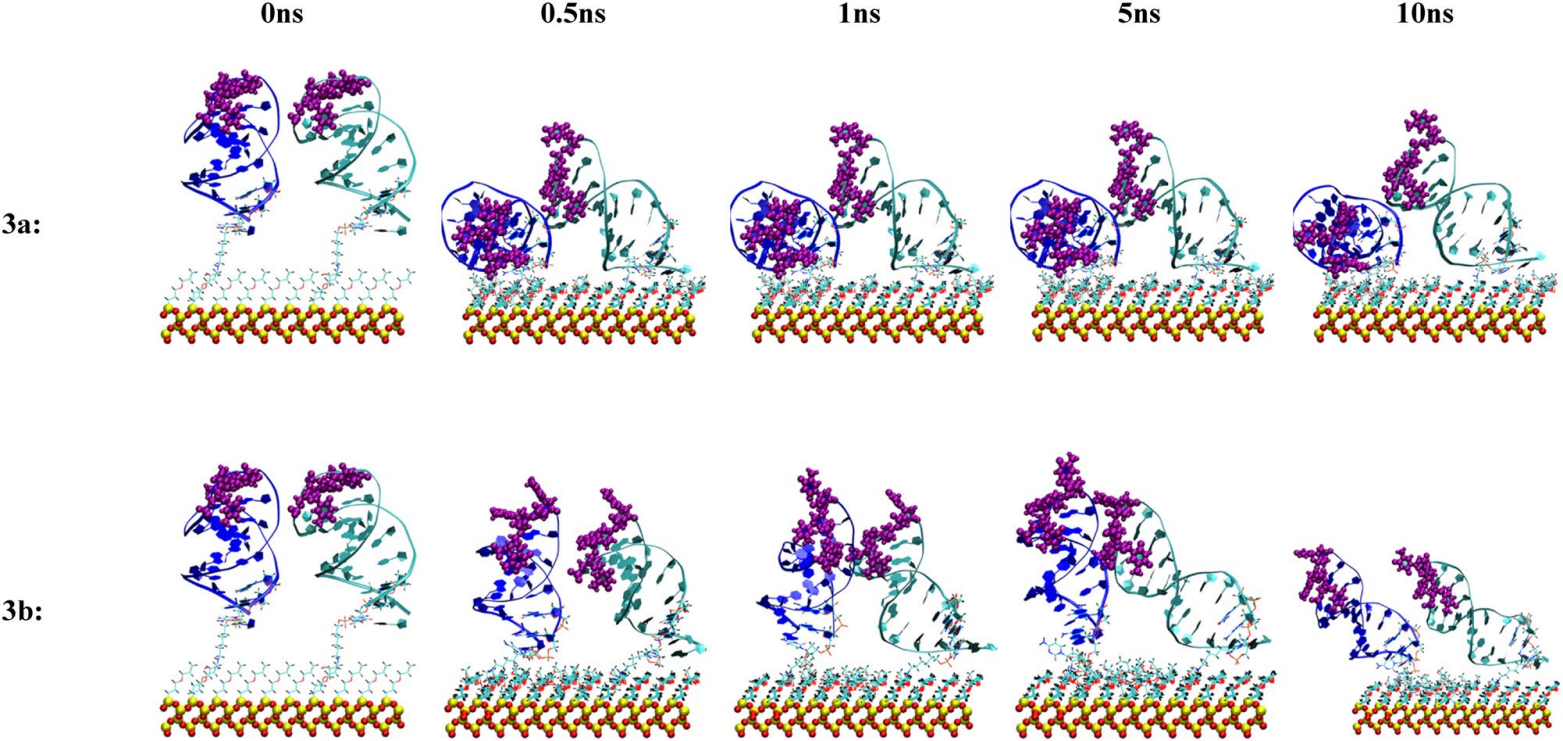
Snapshots of the MD simulation of two anti MUC1 aptamer tethered to the SiO_2_ biosensor substrate in configuration 3 (5′ end attachment, perpendicular to surface, high density) neutralized (top) and in 0.8 M (bottom) solution concentrations at 0 ns, 0.5 ns, 1 ns, 5 ns, and 10 ns. The MUC1 binding residues (thymine residues 11 and 13) are displayed in purple. In each image, aptamer strand 1 is shown on the left and strand 2 is shown on the right.

**Figure 11:**
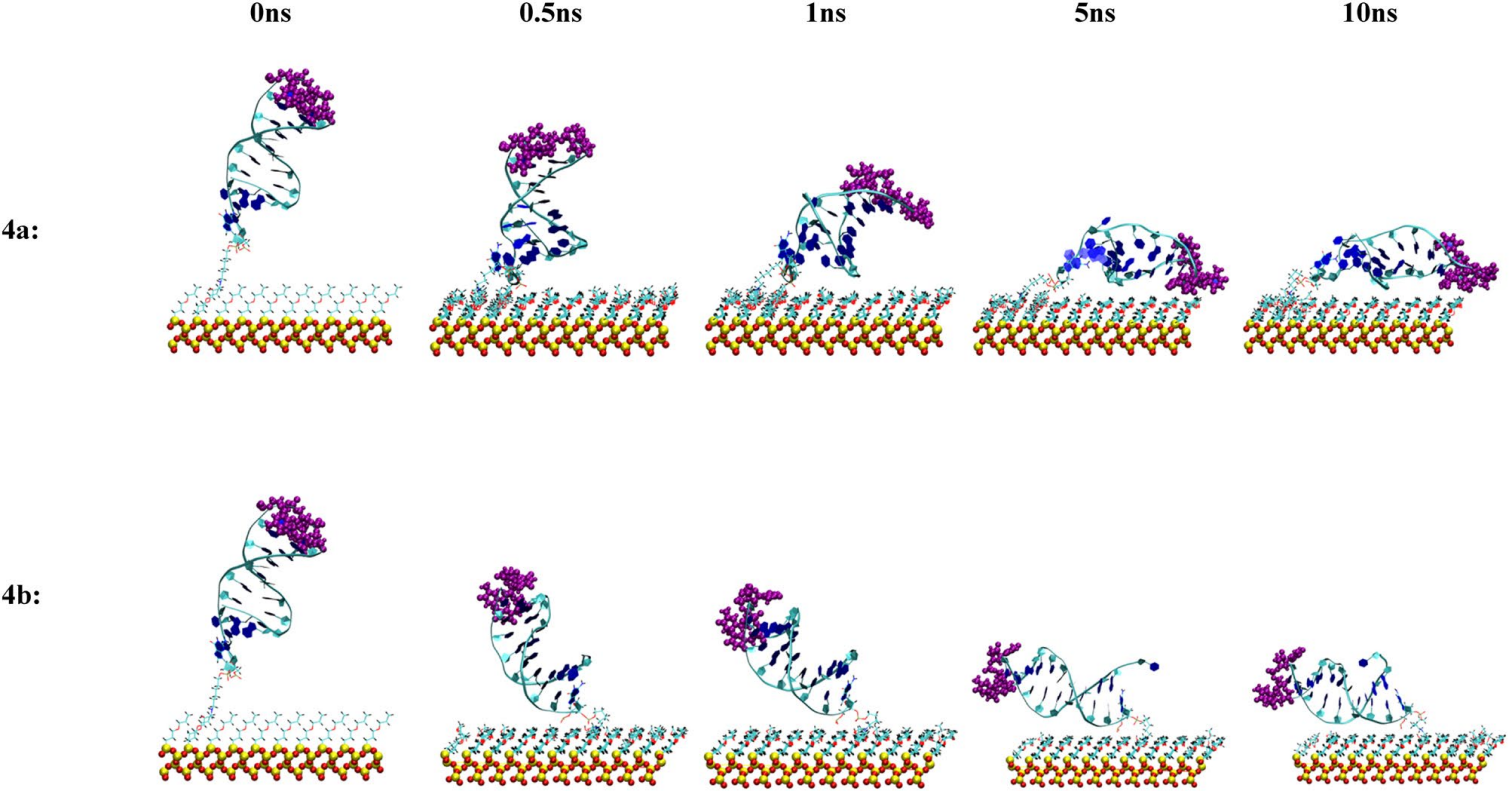
Snapshots of the MD simulation of the anti MUC1 aptamer tethered to the SiO_2_ biosensor substrate in configuration 4 (3′ end attachment, perpendicular to surface, low density) neutralized (top) and in 0.8 M (bottom) solution concentrations at 0 ns, 0.5 ns, 1 ns, 5 ns, and 10 ns. The MUC1 binding residues (thymine residues 11 and 13) are displayed in purple.

**Figure 12:**
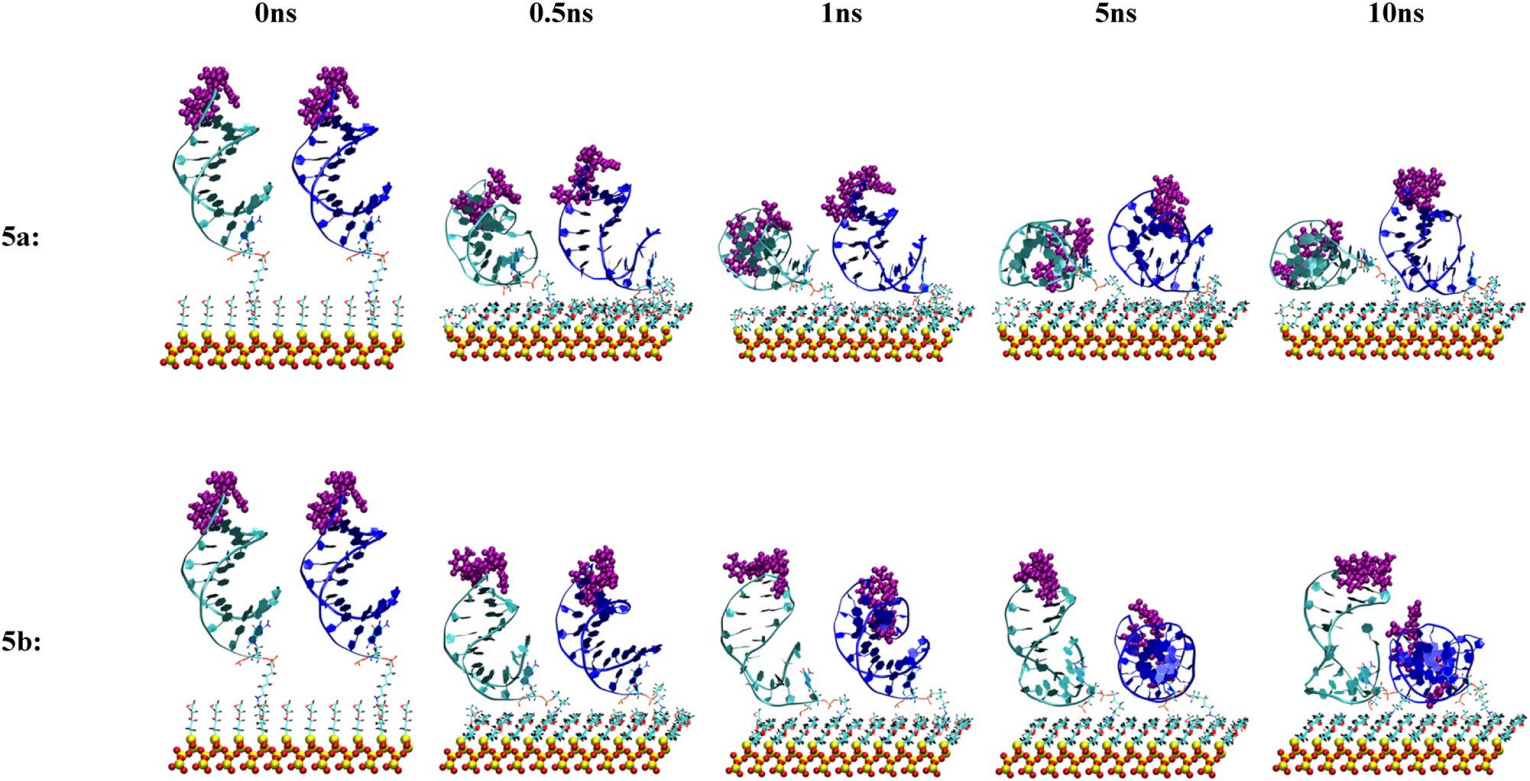
Snapshots of the MD simulation of the anti MUC1 aptamer tethered to the SiO_2_ biosensor substrate in configuration 5 (3′ end attachment, perpendicular to surface, high density) neutralized (top) and in 0.8 M (bottom) solution concentrations at 0 ns, 0.5 ns, 1 ns, 5 ns, and 10 ns. The MUC1 binding residues (thymine residues 11 and 13) are displayed in purple. In each image, aptamer strand 1 is shown on the left and aptamer strand 2 is shown on the right.

Although different methods are available to experimentally determine the density of immobilized aptamers on the biosensor substrate, the aptamer orientation with respect to the biosensor surface so far has evaded experimental measurements. Therefore, it is important to characterize these quantities to obtain molecular insights that are not available experimentally. The orientation of the attached aptamers in the different configurations were visually assessed during the 10 ns MD simulations. Snapshots of the MD simulations of the aptamer tethered to the biosensor substrate for all five configurations in both the neutralized and 0.8 M solution concentrations are displayed at 0 ns, 0.5 ns, 1 ns, and 10 ns time points in Figs. 8, 9, 10, 11 and 12. The viewpoint chosen in each of these figures was selected to enhance visualization of important features. Table 3 contains a summary of the results. The tilt angles with respect to the substrate surface were calculated using VMD^22^ analysis tools.

**Table 3:** Aptamer orientation—summary of findings.

Configuration	Aptamer Strand	Summary of findings
Configuration 1a	1	Collapsed onto surface
Configuration 1b	1	Collapsed onto surface
Configuration 2a	1	Collapsed onto surface
Configuration 2b	1	Stabilized to upright tilted orientation
Configuration 3a	1	Collapsed onto surface
	2	Stabilized to upright tilted orientation but binding site partially blocked
Configuration 3b	1	Stabilized to upright tilted orientation
	2	Stabilized to upright tilted orientation
Configuration 4a	1	Collapsed onto surface
Configuration 4b	1	Collapsed onto surface
Configuration 5a	1	Collapsed onto surface
	2	Collapsed onto surface
Configuration 5b	1	Stabilized to upright tilted orientation
	2	Collapsed onto surface

With the exception of configuration 2b (5′ attachment, perpendicular to surface, low density, 0.8 M solution), shown in Fig. 9, and configuration 3b (5′ attachment, perpendicular to surface, high density, 0.8 M solution), shown in Fig. 10, all other configurations led to the aptamer collapsing onto the biosensor substrate during the course of the 10 ns simulation, limiting access to the active thymine loop (i.e., binding site) of the aptamer and leading to steric hindrance of the binding sites of the aptamer by the SiO_2_ substrate coating.

Configuration 2b stabilized to an upright tilted position with a tilt angle of approximately 70 degrees relative to the substrate after 1 ns and maintained this orientation throughout the remaining 9 ns of the simulation. In this orientation, the active thymine loop (i.e., binding site) of the aptamer was fully exposed and uninhibited by the substrate or neighboring aptamer molecules.

In configuration 3a, shown in Fig. 10, one of the two attached aptamers stabilized to an upright titled position of 45 degrees relative to the substrate at 0.5 ns but the active thymine loop of the aptamer was partially inhibited by the second aptamer, which collapsed onto the substrate after 0.5 ns.

Both attached aptamers in configuration 3b, shown in Fig. 10, stabilized to an upright tilted position of 45 degrees relative to the substrate at 8 ns and maintained this orientation throughout the remaining 2 ns of the simulation. In this orientation, the active thymine loop of the aptamer was fully exposed.

In configuration 5b, shown in Fig. 12, one of the two attached aptamers stabilized to an upright position at 1 ns and maintained this orientation throughout the remaining 9 ns of the simulation; however, the second attached aptamer collapsed onto the substrate after 2 ns.

### Aptamer backbone fluctuations and conformation during the MD simulations

In order to measure the degree of variation of the surface immobilized aptamers from their starting conformation during the 10 ns MD simulation, the Root Mean Square Deviation (RMSD) for each structure was calculated as shown in Supporting Information Figs. S1 to S7.

Table 4 contains the average RMSD and standard deviation for each configuration over the course of the 10 ns MD simulation and the average RMSD and standard deviation for each configuration during the final 2 ns of the MD simulation (i.e., when the aptamers have stabilized). With only two exceptions, the aptamers solvated in the 0.8 M solution had lower RMSD and displayed less variation during the 10 ns simulation than the neutralized aptamers. These two exceptions were limited to one of the two strands in each of the two higher surface density configurations (i.e., aptamer strand 1 in configuration 3a and aptamer strand 1 in configuration 5a). This is attributed to the fact that both these aptamer strands in the neutralized system collapsed (within the first 1 ns of the MD simulation) onto the surface while the corresponding strands stabilized to an upright orientation in the 0.8 M solutions. In a collapsed orientation, the increased interactions with the surface lead to less overall fluctuations (and lower RMSD) as compared to an upright orientation.

**Table 4:** Average RMSD and standard deviation in parenthesis for the nucleic acid backbone of the tethered aptamer in each configuration during the course of the 10 ns MD simulation and the final 2 ns of the MD simulation.

Description	Aptamer Strand	RMSD_10ns_ (Å)	RMSD_2ns_ (Å)
Configuration 1a	1	3.8 (0.8)	4.7 (0.3)
Configuration 1b	1	2.8 (0.5)	3.1 (0.3)
Configuration 2a	1	4.3 (0.7)	4.6 (0.2)
Configuration 2b	1	3.8 (0.6)	4.0 (0.2)
Configuration 3a	1	2.6 (0.5)	2.9 (0.3)
Configuration 3b	1	2.7 (0.5)	2.7 (0.2)
Configuration 3a	2	4.2 (0.8)	4.5 (0.3)
Configuration 3b	2	3.0 (0.5)	3.0 (0.2)
Configuration 4a	1	4.9 (1.1)	5.5 (0.2)
Configuration 4b	1	3.4 (0.6)	3.3 (0.3)
Configuration 5a	1	4.0 (1.0)	4.6 (0.3)
Configuration 5b	1	4.1 (0.8)	4.8 (0.2)
Configuration 5a	2	3.7 (0.7)	4.5 (0.3)
Configuration 5b	2	3.2 (0.6)	3.9 (0.2)

Of the configurations which stabilized to an upright configuration (2b, 3a strand 2, 3b stands 1 and 2, and 5a strand 1), the RMSD for both strands of configuration 3b were markedly lower than the other configurations.

## Conclusions

Herein, we have presented the first computational approach for determining the preferred configuration of an aptamer-based biosensing element attached to a surface. We used all-atom MD simulation studies to investigate the orientation and conformation of single-stranded DNA hairpin aptamers immobilized on biosensor surfaces under a wide variety of conditions. Explicitly, we designed experiments based on the combinations of several variables, including the terminal end of the aptamer used for attachment to the substrate, the surface density of the immobilized aptamers, and the molarity of the solution. Based on the results of our MD studies, we predict that the anti MUC1 aptamer attached at the 5′ end in a high-density configuration to a SiO_2_ substrate and solvated in a 0.8 M solution (i.e., configuration 3b) will exhibit the most enhanced biosensor performance. This is based on (a) the upright orientation of the aptamer resulting in the maximum exposure of the active thymine loop (i.e., binding site) of the aptamer during the MD simulation and (b) the lowest RMSD indicating the least amount of backbone fluctuation and conformational change of the aptamer during the duration of the MD simulation. In comparison, the anti MUC1 aptamer attached at the 5′ end in a low-density configuration to the SiO_2_ substrate and solvated in a 0.8 M solution (i.e., configuration 2b) also stabilized to an upright titled configuration with full exposure of the aptamer binding sites; however, this configuration exhibited higher backbone fluctuations compared to configuration 3b. A possible area of future exploration is to conduct comparative MD studies of the 2b and 3b configurations in the presence of the MUC1 peptide to investigate the aptamer-protein interactions of the immobilized aptamers in these two configurations.

Another interesting observation is the immediate collapse of the anti MUC1 aptamer attached at the 5′ end in the parallel starting orientation (i.e., configuration 1) onto the biosensors substrate. This finding exemplifies the importance of the aptamer immobilization method on biosensor performance and highlights the need for more advanced immobilization techniques which consistently produce an upright starting orientation for the attached aptamer.

Our approach is amenable of experimental verification and it is expected to be applicable not only to other aptamers, such as the more commonly used thrombin and vascular endothelial growth factor (VEGF) aptamers^35^, but also to other surfaces, such as electrochemical biosensor devices’ and nano-delivery systems’ surfaces^20,36^. Combining our approach with simulations of target binding to the aptamers (such as those of Refs. ^13^ and ^14^ for the anti-MUC1 aptamer) can shade further light into the crucial topic of aptamer immobilization effects on their target capture capabilities.

## Supplementary Information

Supplementary Information.
