# Supplementary material for: Computational design of single-stranded DNA hairpin aptamers immobilized on a biosensor substrate
Source: Sci Rep. 2021 May 26;11:10984. doi: 10.1038/s41598-021-88796-2 (PMC8155018; doi:10.1038/s41598-021-88796-2)
Supplement: Supplementary file 1 — Supplementary Information. [file 41598_2021_88796_MOESM1_ESM.pdf]

Supporting Information on: Computational Design of Single-Stranded DNA Hairpin Aptamers Immobilized on a Biosensor Substrate

Iman Jeddi[1] and Leonor Saiz[1,*]

[1]Modeling of Biological Networks and Systems Therapeutics Laboratory, Department of Biomedical Engineering, University of California, 451 East Health Sciences Drive, Davis, CA 95616, USA.

Topology Files

```
*>>>>>> Topology File for SiO2 <<<<<<
27         1

MASS     1 SI              28.08550          SI ! SI
MASS     2 OZ              15.99940          O ! O

MASS    1 SI1              28.08550          SI
MASS    2 O11              15.99940          OZ
MASS    3 SI2              28.08550          SI
MASS    4 O12              15.99940          OZ
MASS    5 SI3              28.08550          SI
MASS    6 O13              15.99940          OZ
MASS    7 SI4              28.08550          SI
MASS    8 O14              15.99940          OZ
MASS    9 O21              15.99940          OZ
MASS   10 O22              15.99940          OZ
MASS   11 O24              15.99940          OZ

AUTO ANGLES DIHE
RESI SIO2    0.00
GROUP
ATOM SI1          SI          1.0
ATOM O11          OZ         -0.5
ATOM SI2          SI          1.0
```

* To whom correspondence should be addressed: lsaiz@ucdavis.edu

```
ATOM O12        OZ       -0.5
ATOM SI3        SI        1.0
ATOM O13        OZ       -0.5
ATOM SI4        SI        1.0
ATOM O14        OZ       -0.5
ATOM O21        OZ       -0.5
ATOM O22        OZ       -0.5
ATOM O24        OZ       -0.5
BOND SI1 O21  SI1 O11  SI2 O22
BOND SI2 O24  SI2 O12  SI2 O13  SI3 O21 BOND SI3 O13  SI4 O22  SI4 O14

END
```

```
*>>>>>> Topology File for Epoxide Monolayer  <<<<<<
27          1

MASS     1 C          12.01070            C
MASS     1 H2          1.00794            H
MASS     1 H3          1.00794            H
MASS     1 C2         12.01070            C
MASS     1 H4          1.00794            H
MASS     1 C3        12.01070             C
MASS     1 H5          1.00794            H
MASS     1 H6          1.00794            H
MASS     1 H7          1.00794            H
MASS     1 O         15.99940             O
MASS     1 C4        12.01070             C
MASS     1 C5        12.01070             C
MASS     1 H8          1.00794            H
MASS     1 H9         1.00794             H
MASS     1 O2        15.99940             O
MASS     1 C6        12.01070             C
MASS     1 H10         1.00794            H
MASS     1 H11         1.00794            H
MASS     1 H12         1.00794            H

AUTO ANGLES DIHE
RESI  MET        0.00
GROUP
ATOM   C      CG321           -0.270
ATOM   H2    HGA2              0.090
ATOM   H3    HGA2              0.090
ATOM   C2    CG321            -0.180
ATOM   H4    HGA2              0.090
ATOM   C3    CG321            -0.010
ATOM   H5    HGA2              0.090
ATOM   H6    HGA2              0.090
ATOM   H7    HGA2              0.090
ATOM   O     OG301            -0.340
ATOM   C4    CG3C31            0.110
ATOM   C5    CG321            -0.010
ATOM   H8    HGA2              0.090
ATOM   H9    HGA2              0.090
ATOM   O2    OG3C31          -0.400
ATOM   C6    CG3C31           0.020
ATOM  H10  HGA1               0.090
ATOM  H11  HGA2               0.090
ATOM  H12  HGA2               0.090
BOND C H2   C H3
BOND C C2    C2 H4  C2 C3  C2 H5
BOND C3 H6  C3 H7  C3 O   O  C5
BOND C4 C5  C4 O2  C4 H10 C4 C6
BOND C5 H9  C5 H8  O2 C6  C6 H11
BOND C6 H12

END
```

*>>>>>> **Topology File for Epoxide Amine Linker** <<<<<<

```
27        1

MASS     1 P        30.97376  P
MASS     1 O1P      15.99940  O
MASS     1 O2P      15.99940  O
MASS     1 O5'      15.99940  O
MASS     1 C5'      12.01070  C
MASS     1 H5'       1.00794  H
MASS     1 H5''      1.00794  H
MASS     1 O3'      15.99940  O
MASS     1 C        12.01070  C
MASS     1 H2        1.00794  H
MASS     1 H3        1.00794  H
MASS     1 C2       12.01070  C
MASS     1 H4        1.00794  H
MASS     1 H5        1.00794  H
MASS     1 C3       12.01070  C
MASS     1 H6        1.00794  H
MASS     1 O        15.99940  O
MASS     1 H7        1.00794  H
MASS     1 C4       12.01070  C
MASS     1 H8        1.00794  H
MASS     1 H9        1.00794  H
MASS     1 C5       12.01070  C
MASS     1 O2       15.99940  O
MASS     1 C6       12.01070  C
MASS     1 H10       1.00794  H
MASS     1 H11       1.00794  H
MASS     1 H12       1.00794  H
MASS     1 N        14.00670  N
MASS     1 H13       1.00794  H
MASS     1 H14       1.00794  H
MASS     1 C7       12.01070  C
MASS     1 H15       1.00794  H
MASS     1 C8       12.01070  C
MASS     1 H16       1.00794  H
MASS     1 C9       12.01070  C
MASS     1 H17       1.00794  H
MASS     1 H18       1.00794  H
MASS     1 H19       1.00794  H
MASS     1 H20       1.00794  H
MASS     1 C10      12.01070  C
MASS     1 C11      12.01070  C
MASS     1 H21       1.00794  H
MASS     1 H22       1.00794  H
MASS     1 H23       1.00794  H
MASS     1 H24       1.00794  H
MASS     1 C12      12.01070  C
MASS     1 H26       1.00794  H
MASS     1 H27       1.00794  H
```

```
AUTO ANGLES DIHE

RESI  MEZ     0.00
GROUP
ATOM   P            P           1.50000
ATOM  O1P          ON3      -0.78000
ATOM  O2P          ON3      -0.78000
ATOM  O5'          ON2      -0.57000
ATOM  C5'          CN8B    -0.08000
ATOM  H5'          HN8       0.09000
ATOM  H5''         HN8       0.09000
ATOM  O3'          ON2      -0.57000
ATOM  C            CG321  -0.27000
ATOM  H2           HGA2     0.09000
ATOM  H3           HGA2     0.09000
ATOM  C2           CG321  -0.18000
ATOM  H4           HGA2     0.09000
ATOM  H5           HGA2     0.09000
ATOM  C3           CG321  -0.01000
ATOM  H6           HGA2     0.09000
ATOM  O            OG301  -0.34000
ATOM  H7           HGA2     0.09000
ATOM  C4           CG321  -0.01000
ATOM  H8           HGA2     0.09000
ATOM  H9           HGA2     0.09000
ATOM  C5           CG321  -0.14000
ATOM  O2           OG311 -0.65000
ATOM  C6           CG321  -0.06000
ATOM  H10          HGA2     0.09000
ATOM  H11          HGA2     0.09000
ATOM  H12          HGA2     0.09000
ATOM  N            NG311 -0.82000
ATOM  H13          HGP1     0.42000
ATOM  H14          HGP1     0.40000
ATOM  C7           CG321  -0.06000
ATOM  H15          HGA2     0.09000
ATOM  C8           CG321  -0.18000
ATOM  H16          HGA2     0.09000
ATOM  C9           CG321  -0.18000
ATOM  H17          HGA2     0.09000
ATOM  H18          HGA2     0.09000
ATOM  H19          HGA2     0.09000
ATOM  H20          HGA2     0.09000
ATOM  C10          CG321  -0.18000
ATOM  C11          CG321  -0.18000
ATOM  H21          HGA2     0.09000
ATOM  H22          HGA2     0.09000
ATOM  H23          HGA2     0.09000
ATOM  H24          HGA2     0.09000
ATOM  C12          CG321  -0.08000
ATOM  H26          HGA2     0.09000
ATOM  H27          HGA2     0.09000
```

```
BOND P O3'  P O2P  P O5'
BOND P O1P  O5' C5'  C5' H5''  C5' H5'
BOND O3' C12  C H2  C C2  C H3
BOND C2 H5  C2 H4  C2 C3  C3 H7
BOND C3 H6  C3 O  O C4  C4 H9
BOND C4 C5  C4 H8  C5 O2  C5 H10
BOND C5 C6  O2 H13  C6 H12  C6 N
BOND C6 H11  N C7  N H14  C7 H15
BOND C7 H16  C7 C8  C8 H17  C8 C9
BOND C8 H18  C9 H19  C9 C10  C9 H20
BOND C10 H21  C10 H22  C10 C11  C11 H23
BOND C11 C12  C11 H24  C12 H27  C12 H26


END
```

Additional Force Field Parameters

BONDS

```
CG321  ON2    320.0     1.44   ! Adapted from CN8B/ON2
```

ANGLES

```
CG321    ON2     P          20.0      120.0   35.00   2.33     !Adapted from CN8B/ON2/P
CG321    CG321  ON2       45.00     111.50                     ! Adapted from CG321/CG321/OG301
OG301    CG321  OG311    75.70     110.10                      ! Adapted from CG321/CG321/OG311
ON2      CG321  HGA2      45.90     108.89                     ! Adapted from OG301/CG321/HGA2
CG3C31  CG321  OG301    95.00     109.70                       ! Adapted from CG321/OG301/CG321
```

DIHEDRALS

```
OG311 CG321  CG321  NG311   0.06450 2     0.00 ! Adapted from CG321/CG321/CG321/CG321
CG321  CG321  CG321  NG311    0.06450 2     0.00 ! Adapted from CG321/CG321/CG321/CG321
CG321  CG321  NG311 CG321     0.06450 2     0.00 ! Adapted from CG321/CG321/CG321/CG321
CG321  CG321  ON2     P          0.2        1  120.0   ! Adapted from CN7/CN8B/ON2/P
P        ON2     CG321   HGA2     0.000     3      0.0   ! Adapted from P/ON2/CN8B/HN8
P        ON2     CG321   HGA2     0.000     3      0.0   ! Adapted from P/ON2/CN8B/HN8
ON3     P    ON2    CG321    0.10    3      0.0           ! Adapted from ON3/P/PN2/CN8B
ON2     P    ON2    CG321    1.20    1   180.0            ! Adapted from ON2/P/PN2/CN8B
ON2     P    ON2    CG321    0.10    2   180.0            ! Adapted from ON2/P/PN2/CN8B
ON2     P    ON2    CG321    0.10    3   180.0            ! Adapted from ON2/P/PN2/CN8B
ON2     P    ON2    CG321    0.00    6      0.0           ! Adapted from ON2/P/PN2/CN8B
CG321  OG301  CG321 CG3C31  0.06450 2    0.00 ! Adapted from CG321/CG321/CG321/CG321
CG321  OG301  CG321 CG3C31  0.14975 3 180.00 ! Adapted from CG321/CG321/CG321/CG321
CG321  OG301  CG321 CG3C31  0.09458 4    0.00 ! Adapted from CG321/CG321/CG321/CG321
CG321  OG301  CG321 CG3C31  0.11251 5    0.00 ! Adapted from CG321/CG321/CG321/CG321
CG321  CG321  CG321  ON2       0.1600  1 180.00 ! Adapted from CG321/CG321/CG321/OG301
CG321  CG321  CG321  ON2       0.3900  2   0.00 ! Adapted from CG321/CG321/CG321/OG301
OG301 CG321  CG321  OG311    0.1950  3   0.00 ! Adapted from CG321/CG321/CG321/OG311
ON2    CG321  CG321  HGA2       0.1900  3   0.00 ! Adapted from OG301/CG321/CG321/HGA2
ON2    CG321  CG321  HGA2       0.1950  3   0.00 ! Adapted from OG301/CG321/CG321/HGA2
OG301 CG321  CG3C31 CG3C31 0.0000  3   0.00  ! Adapted from CG331/CG321/CG3C31/CG3C31
OG301 CG321  CG3C31 OG3C31 0.4700 1 180.00 ! Adapted from CG331/CG321/CG3C31/OG3C31
OG301 CG321  CG3C31 OG3C31 0.1300  3  0.00 ! Adapted from CG331/CG321/CG3C31/OG3C31
OG301 CG321  CG3C31 HGA1    0.1800  1    0.00 ! Adapted from CG331/CG321/CG3C31/HGA1
OG301 CG321  CG3C31 HGA1    1.0700  3    0.00 ! Adapted from CG331/CG321/CG3C31/HGA1
CG321  CG321  NG311  HGP1     0.1000  3    0.00 ! Adapted from CG331/CG321/NG311/HGP1
```

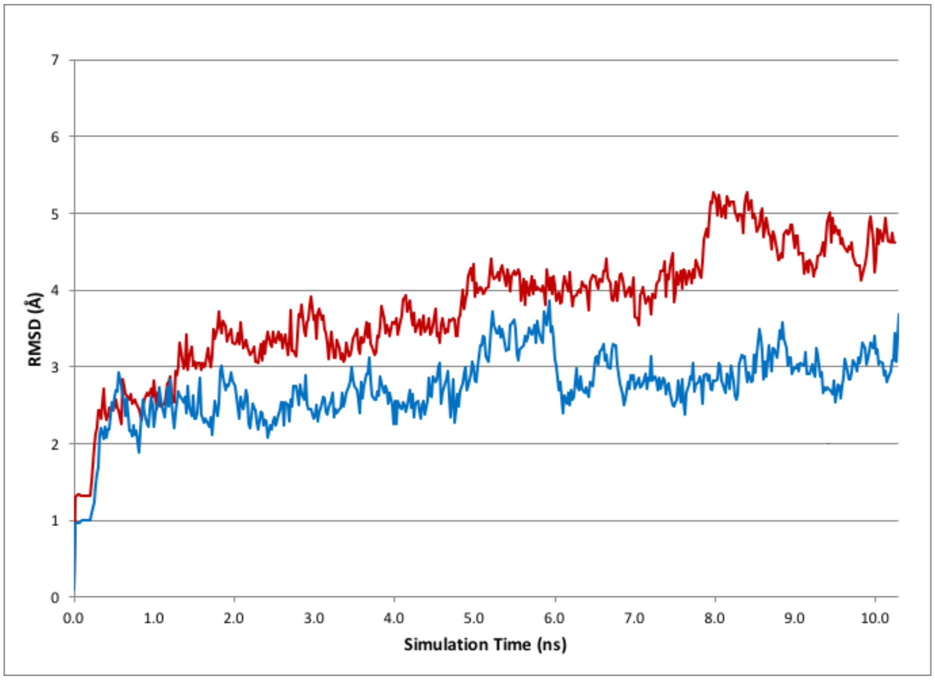

Figure S1: RMSD of the anti MUC1 aptamer as tethered to the SiO_2 biosensor substrate in configuration 1(5' end attachment, parallel to surface, low density) for the neutralized system (red) and 0.8M (blue) solution concentration during the 10ns MD simulation.

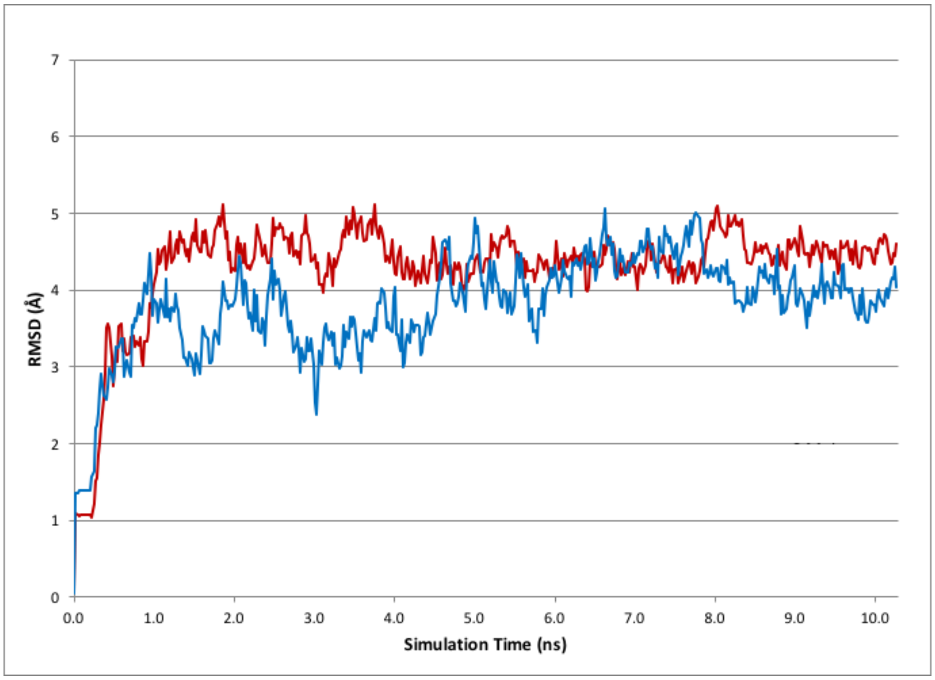

Figure S2: RMSD of the anti MUC1 aptamer in configuration 2 (5' end attachment, perpendicular to surface, low density) for the neutralized system (red) and 0.8M (blue) solution concentration.

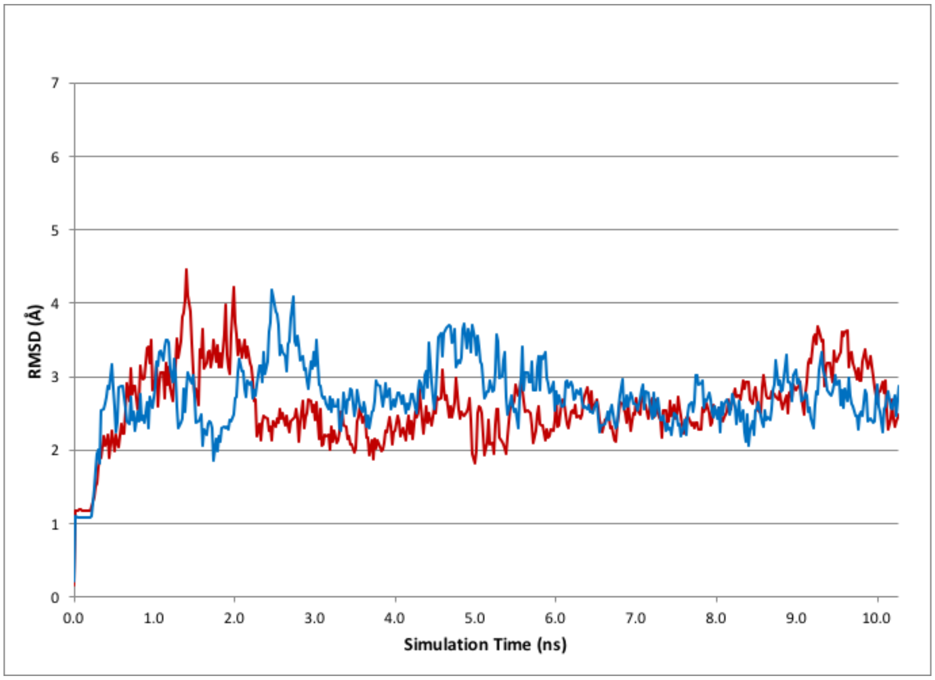

Figure S3: RMSD of the anti MUC1 aptamer strand 1 in configuration 3 (5' end attachment, perpendicular to surface, high density) for the neutralized system (red) and 0.8M (blue) solution concentration.

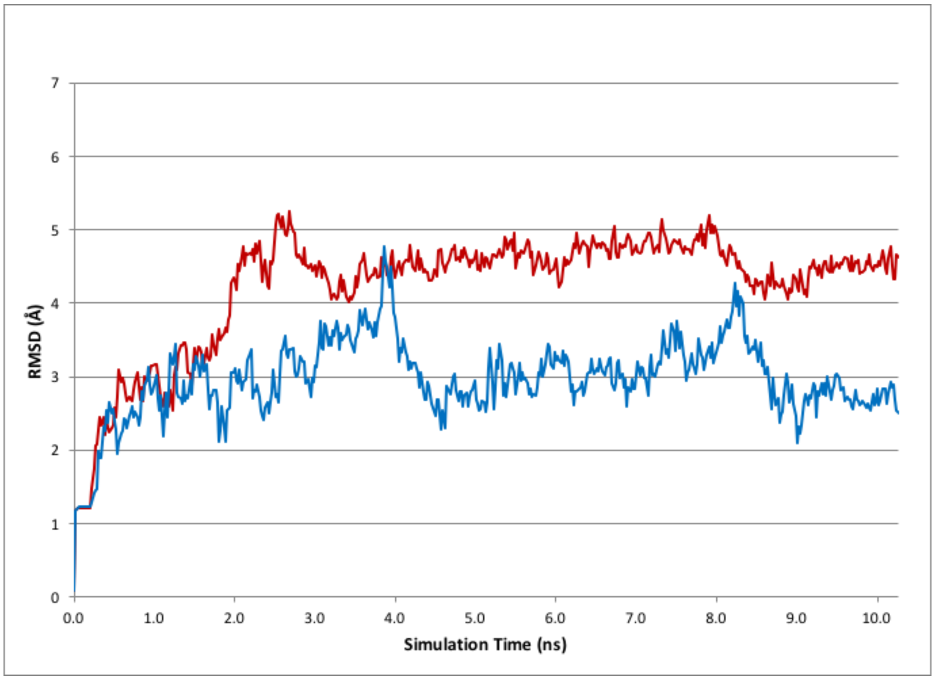

Figure S4: RMSD of the anti MUC1 aptamer strand 2 in configuration 3 (5' end attachment, perpendicular to surface, high density) for the neutralized system (red) and 0.8Molar (blue) solution concentration.

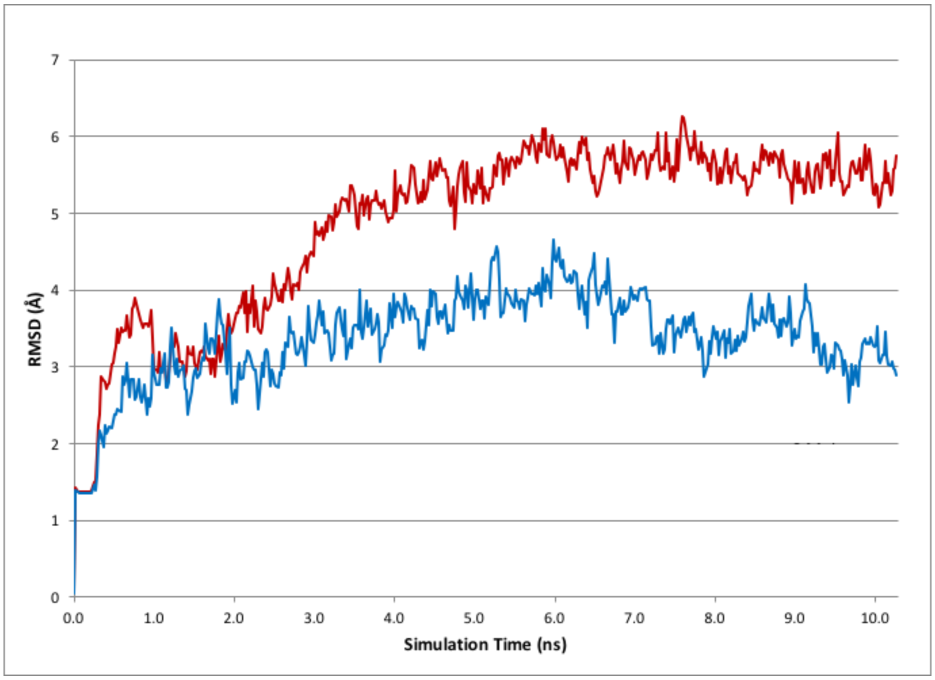

Figure S5: RMSD of the anti MUC1 aptamer in configuration 4 (3' end attachment, perpendicular to surface, low density) for the neutralized system (red) and 0.8M (blue) solution concentration.

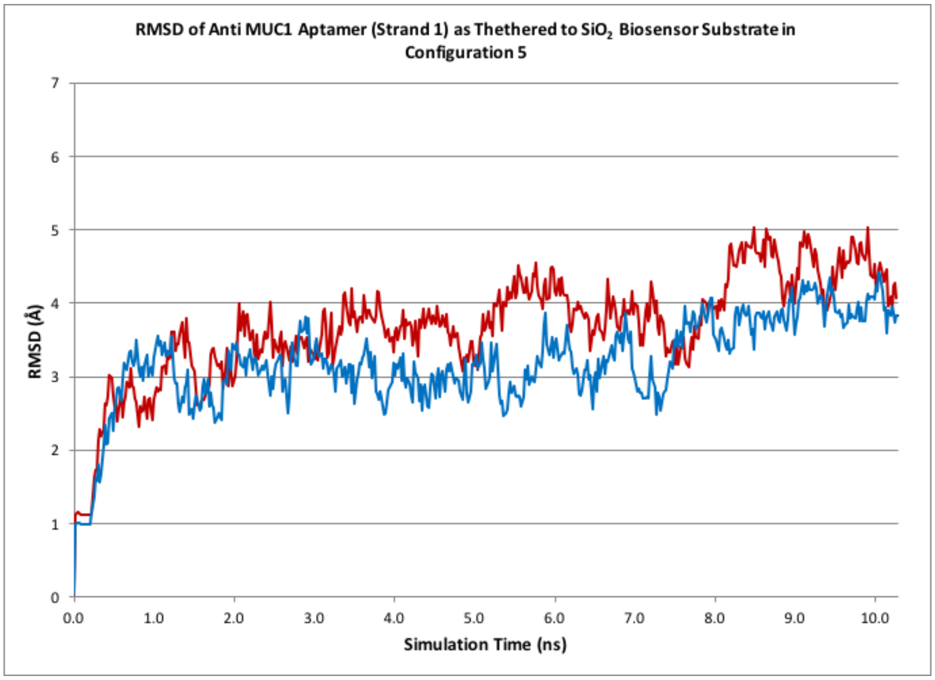

Figure S6: RMSD of the anti MUC1 aptamer strand 1 in configuration 5 (5' end attachment, perpendicular to surface, high density) for the neutralized system (red) and 0.8M (blue) solution concentration.

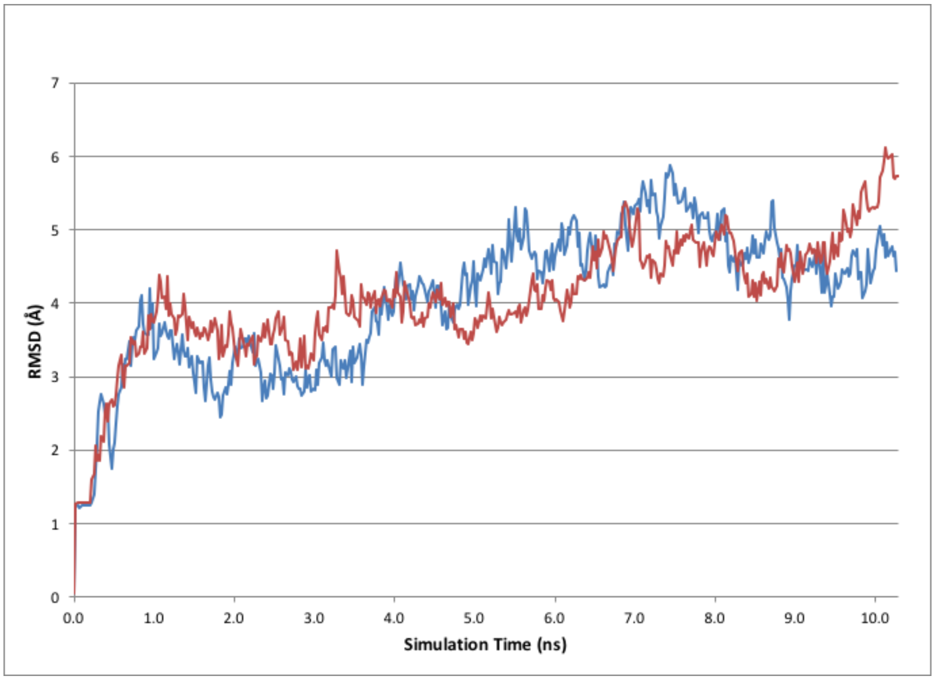

Figure S7: RMSD of anti MUC1 aptamer strand 2 in configuration 5 (5' end attachment, perpendicular to surface, high density) for the neutralized system (red) and 0.8M (blue) solution concentration.
